# A bibliometric analysis reveals a dynamic growth in the use of artificial intelligence in oral cancer research over three decades

**DOI:** 10.1007/s12672-025-03293-6

**Published:** 2025-07-28

**Authors:** Irna Sufiawati, Anisa Insyafiana, Rifat Rahman, Adi Idris

**Affiliations:** 1 Department of Oral Medicine, Faculty of Dentistry, Universitas Padjadjaran, Bandung, West Java Indonesia; 2 Institute for Biomedicine and Glycomics, School of Pharmacy and Medical Sciences, Griffith University, Southport, QLD Australia; 3 Centre for Immunology and Infection Control, School of Biomedical Sciences, Queensland University of Technology, Brisbane, QLD Australia

**Keywords:** Artificial intelligence, Bibliometric analysis, Oral cancer, Research trends, VOSviewer

## Abstract

Oral cancer (OC) remains a significant malignant neoplasm in both the developed and developing world. Artificial intelligence (AI) has had a significant impact on scientific disciplines, including oncology by transforming data analysis and predictive capabilities. Recent advancements in AI have enabled researchers to integrate and synthesize multidimensional datasets, infer patterns, and predict outcomes, ultimately enhancing shared decision-making between patients and clinicians. This bibliometric analysis aims to provide a comprehensive overview of the application of AI in OC research over the last three decades. Our analysis of 351 articles retrieved from SCOPUS between 1998 and 2024 using VOSviewer highlights the dynamic growth of AI in OC research. The significant trends in publications and citations reflect the increasing interest and impact of this field. These findings provide valuable insights for policymakers, funding agencies, and researchers, guiding future efforts to integrate AI technologies into oral oncology practices.

**Supplementary Information:**

The online version contains supplementary material available at 10.1007/s12672-025-03293-6.

## Introduction

Oral cancer (OC) is a major public health concern worldwide and its epidemiology varies widely across populations. According to the International Agency for Research on cancer (IARC), the global incidence of lip cancer and OC is expected to reach 389,846 new cases and 188,438 deaths by 2022 [1]. The increasing number of OC cases globally has driven the development of better technologies for early OC detection to assist doctors in patient management.

Artificial intelligence (AI) has had a significant impact on scientific disciplines, including oncology by transforming data analysis and predictive capabilities. Importantly, AI have enabled researchers to integrate and synthesize multidimensional datasets, infer patterns, and predict outcomes, ultimately enhancing shared decision-making between patients and clinicians [2, 3]. Within the context of OC, AI operates through two primary mechanisms: computational processes for data analysis and deep learning algorithms for enhanced accuracy in diagnostic results [4]. Indeed, the application of AI has progressively expanded in the field of dentistry, specifically in basic oral oncology research [5, 6] and for the early clinical detection of OC [4, 7]. The use of AI in OC research operates in two phases, namely training and then testing the AI model with structured datasets, often derived from patient information or clinical outcomes. The testing phase serves to validate the AI model’s predictive accuracy [8, 9]. Furthermore, studies have shown that combining AI with biomarkers can improve the early detection of malignant lesions thereby advancing diagnostic precision and therapeutic strategies [10, 11]. 

Bibliometric mapping offers a strategic approach to evaluate the current landscape of AI in oral oncology research and to guide future research directions. By systematically analyzing the literature, this approach identifies prevailing research trends and emerging topics. Importantly, it highlights key contributors (e.g., influential authors, institutions) and their collaborative networks and elucidates the thematic evolution of the field over time. Equally important, it highlights potential knowledge gaps in the existing literature, thereby revealing opportunities for new investigations.

This bibliometric analysis aims to provide a comprehensive overview of the application of AI in OC research over the last three decades using the visual mapping method (VOSviewer), from 1998 to 2024. By analyzing data from major scientific databases, we highlight trends, key contributors, and prospects for integrating AI into oral clinical practice.

## Materials and methods

### Data source and search strategy

**Fig. 1:**
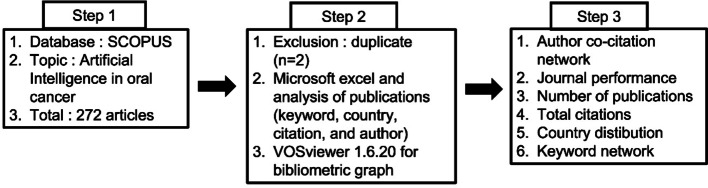
Flow diagram of bibliometric analysis process in this study. Data were extracted from SCOPUS using VOSviewer software, covering the period between 1998 and 2024

Data were retrieved from SCOPUS between 01 January 1998 and December 2024, imported into Microsoft Excel, and analyzed using VOSviewer 1.6.20. Bibliometric parameters such as publication trends, citations, author collaborations, and keyword co-occurrence were analyzed using Microsoft Excel and VOSviewer. VOSviewer facilitates the visualization of associations between affiliated countries and institutions, identifies keywords, assesses the most productive publications, evaluates journal relevance, and gauges author productivity [12, 13]. Duplicate research articles, retracted articles, preprints, non-peer-reviewed articles, conference papers, and those that lacked relevance to the topic were excluded. Only journal articles focused on AI and OC written in English were included in this study. We performed a search using the following criteria: Title-ABS-key (artificial and intelligence and oral and cancer) and (limit-to (exact keyword, “artificial intelligence”) or limit-to (exact keyword, “machine learning”) or limit-to (exact keyword, “oral cancer”) or limit-to (exact keyword, “human”) or limit-to (exact keyword, “deep learning”) or limit-to (exact keyword, “mouth tumor”) or limit-to (exact keyword, “mouth neoplasms,” “diseases”) or limit-to (exact keyword “mouth cancer”) or limit-to (exact keyword, “article”). The overview of the bibliometric process is shown in Fig. 1. Statistical analyses were performed to identify key trends, correlations, and relationships within the data.

## Results

### General data, publication and citation trends

**Fig. 2:**
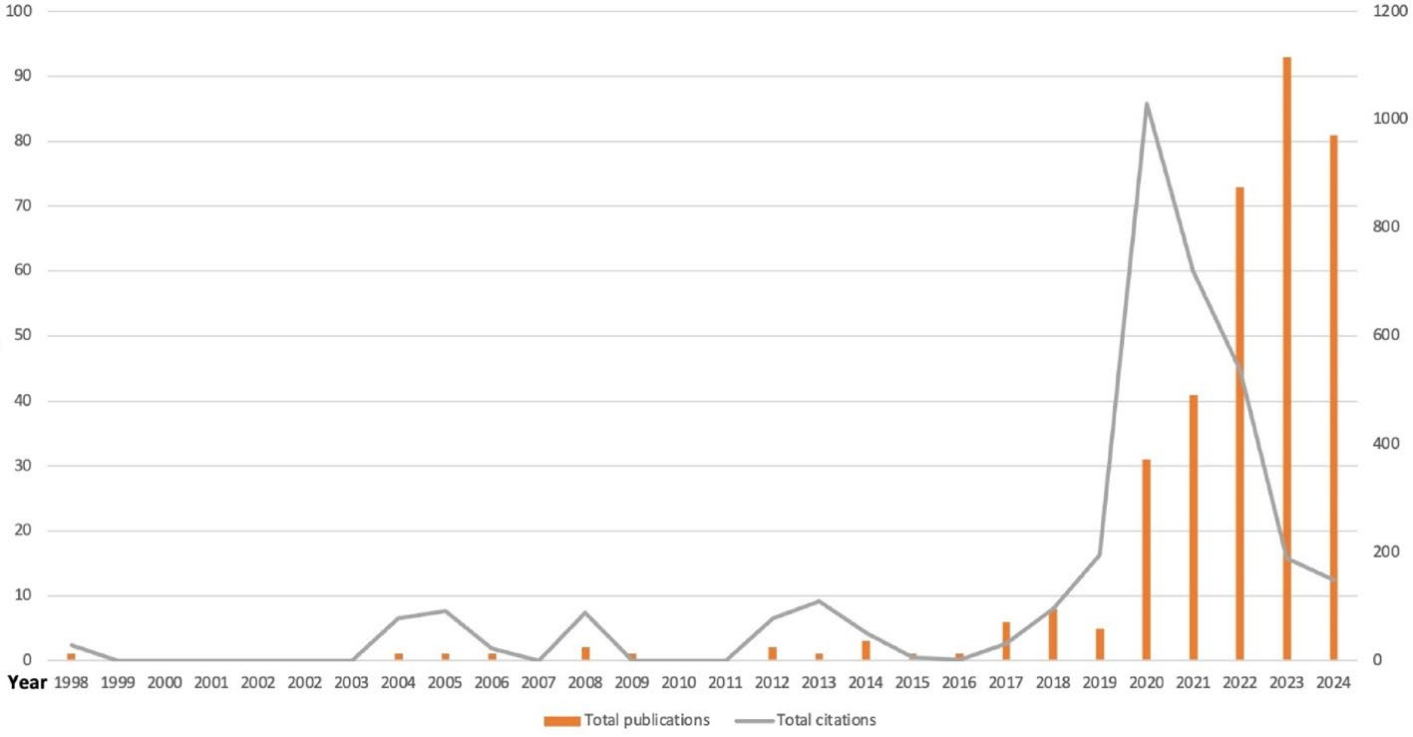
Total number of publications and total citations by year

**Fig. 3:**
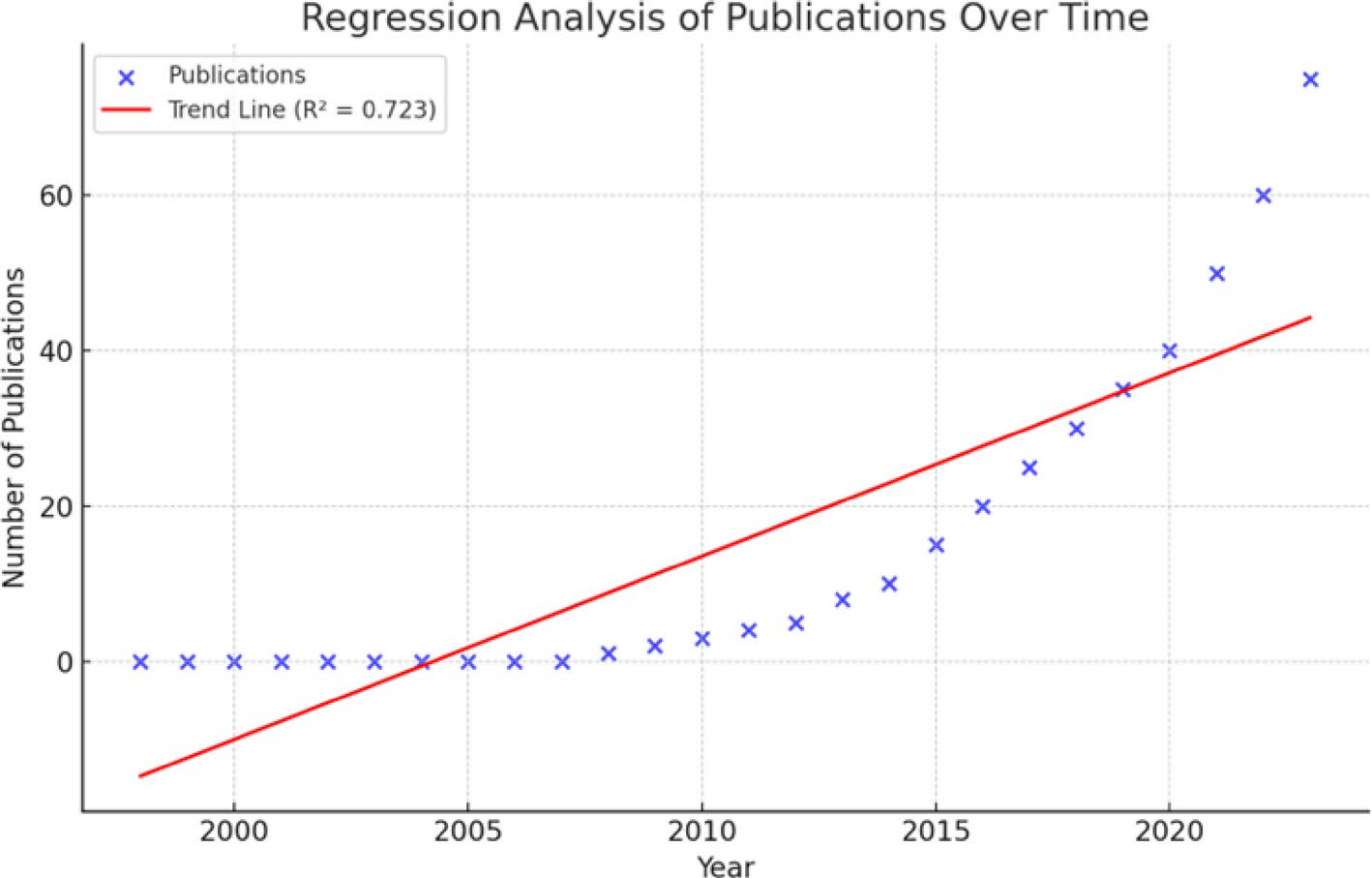
Regression analysis of publications over time

**Fig. 4:**
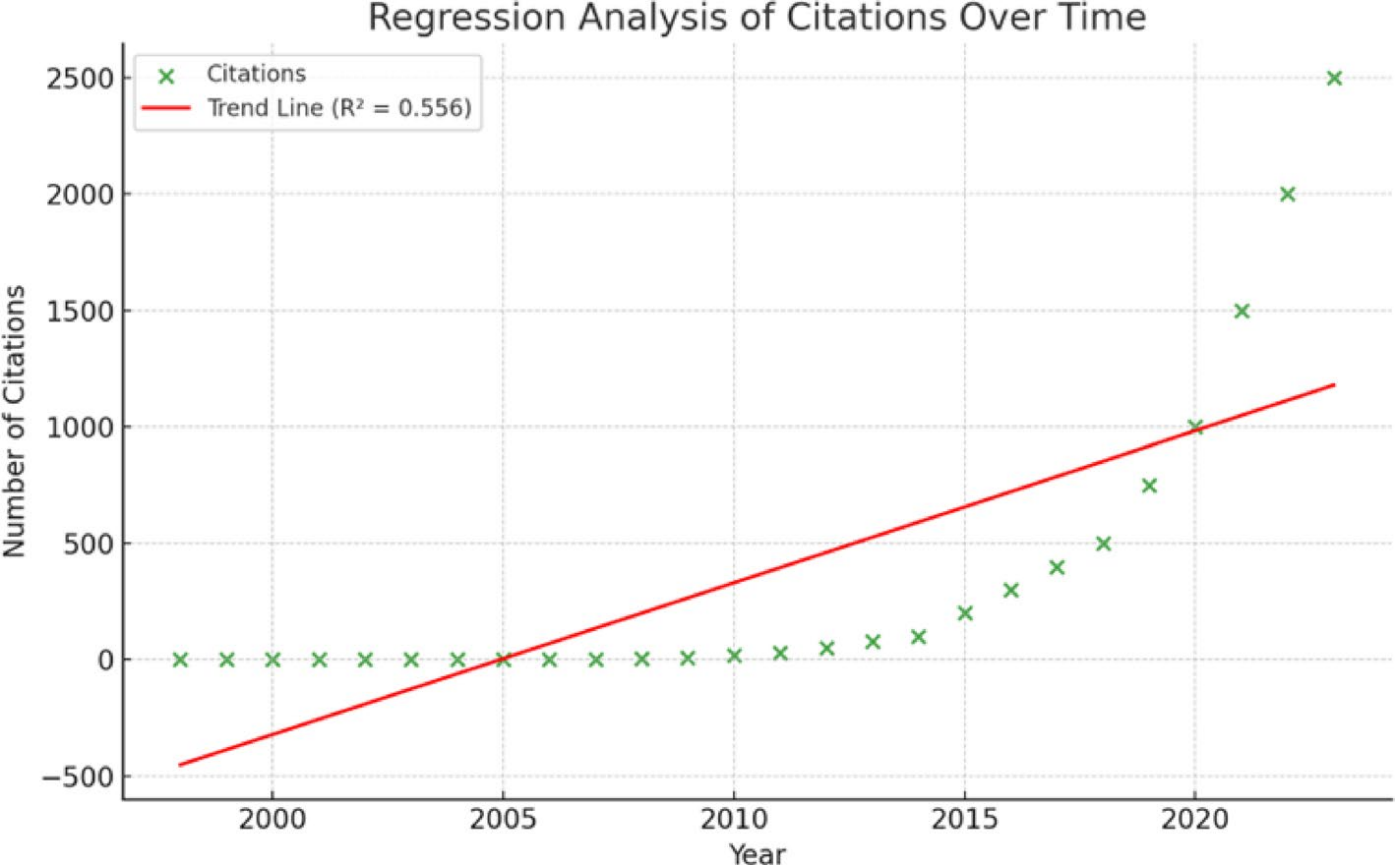
Regression analysis of citations over time

From 1998 to 2024, 351 articles were extracted for analysis; two publications were duplicated and excluded. Although only four articles were published in the first decade, a dramatic increase in the number of publications was observed from 2023 onwards (93 publications), underscoring the emerging interest in AI in OC research (Fig. 2). Next, a linear regression analysis was conducted to assess publication and citation trends over time. Linear regression analysis revealed a significant increase in AI-related publications in OC research over time (Fig. 3). The analysis revealed a statistically significant upward trend (slope = 2.36, R² = 0.723, *p* = 3.86e-08), highlighting a consistent increase in research output in this field. Citations in this field of work also showed a statistically significant upward trend (Fig. 4). Our analysis shows the increasing citation impact of AI research in OC, with an annual growth rate of 65.22 citations. Citations also showed a significant upward trend (slope = 65.22, R² = 0.556, *p* = 1.24e-05) indicating statistical significance (*p* = 1.24e-05). This reflects the growing impact of recent research in this field.

### Author collaborations, institutions, and publishers

**Fig. 5:**
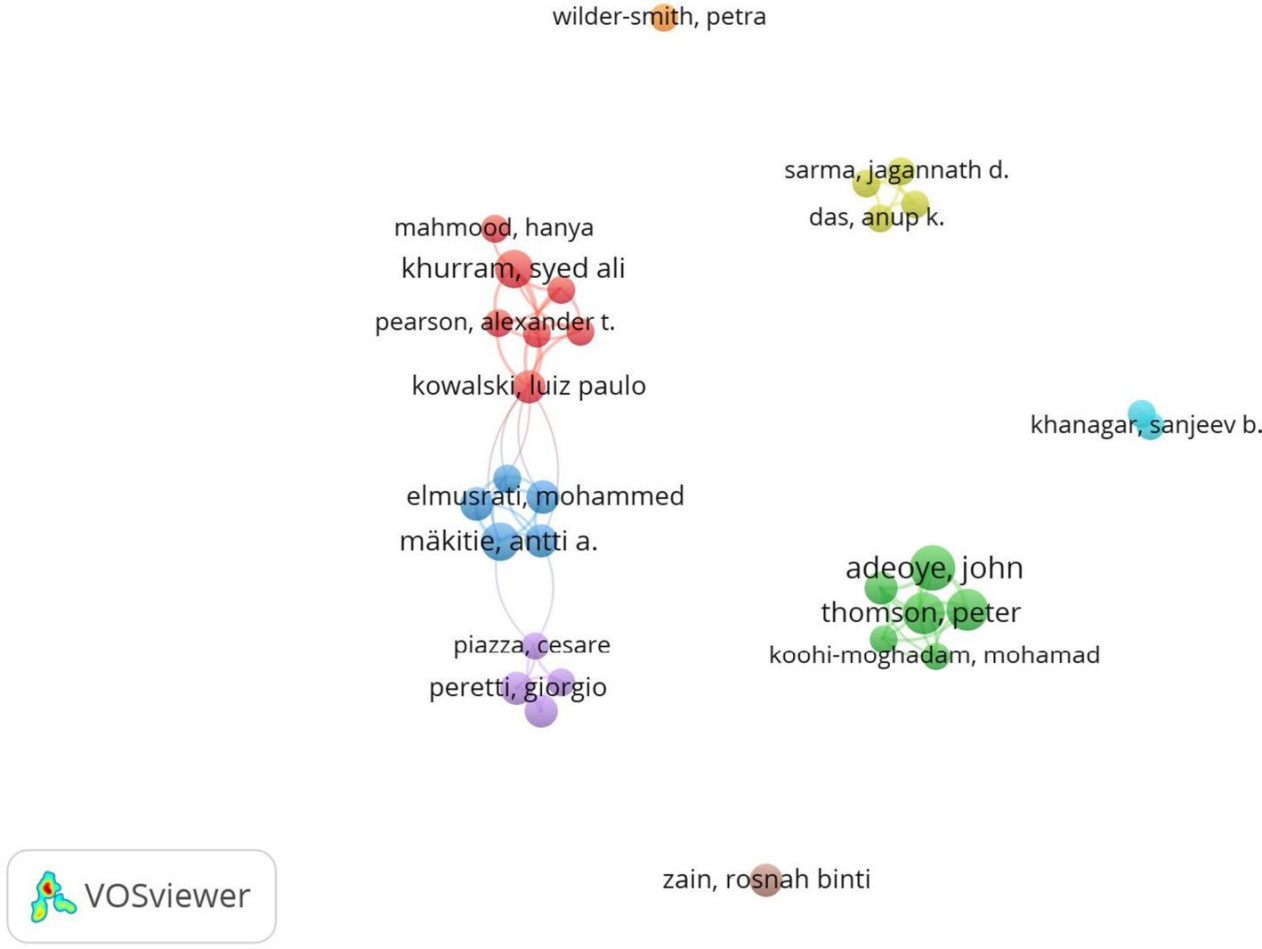
Intellectual structural based on co-authorship

Our analysis shows a cluster of eight author collaboration groups in the field of AI in OC research (Fig. 5). This visualization is representative of only 30 authors and a minimum of five documents per author. Our analysis revealed that 16 authors had strong relationships in the network. Adeoye J had the highest citation value per publication, where seven published articles have been cited a total of 138 times (Table S1). In short, the author collaboration networks reveal several distinct clusters of researchers, suggesting the presence of specialized subgroups within the field of AI in OC. These clusters likely reflect collaborative efforts focused on specific research themes such as image-based diagnostics or machine learning algorithms. Notably, high-impact contributors, such as Adeoye J are positioned centrally within these networks, indicating their pivotal role in knowledge dissemination and cross-institutional collaboration. The clustering pattern also suggests that while there is a growing body of collaborative work, opportunities remain to enhance inter-cluster connections and foster more interdisciplinary and global partnerships in the field of AI in oral oncology.

University of Sheffield and the University of Hong Kong ranked the highest for the number of publications in this field of work. Our analysis also showed that the journal with the most publications in this field of work is *Cancers* (Table S2). There were 19 publications with average citation scores of 8.15. Importantly, *Oral Oncology* has the highest citation per publication (CCP) score (CCP = 22.9). VOSviewer was used to analyze the number of articles cited. Out of the top ten authors who were cited more than 50 times in this field of work, the lead author with the highest number of citations is Almangush et al., (144 citations) (Table S3).

### Analysis of citations by country and geographical region

**Fig. 6:**
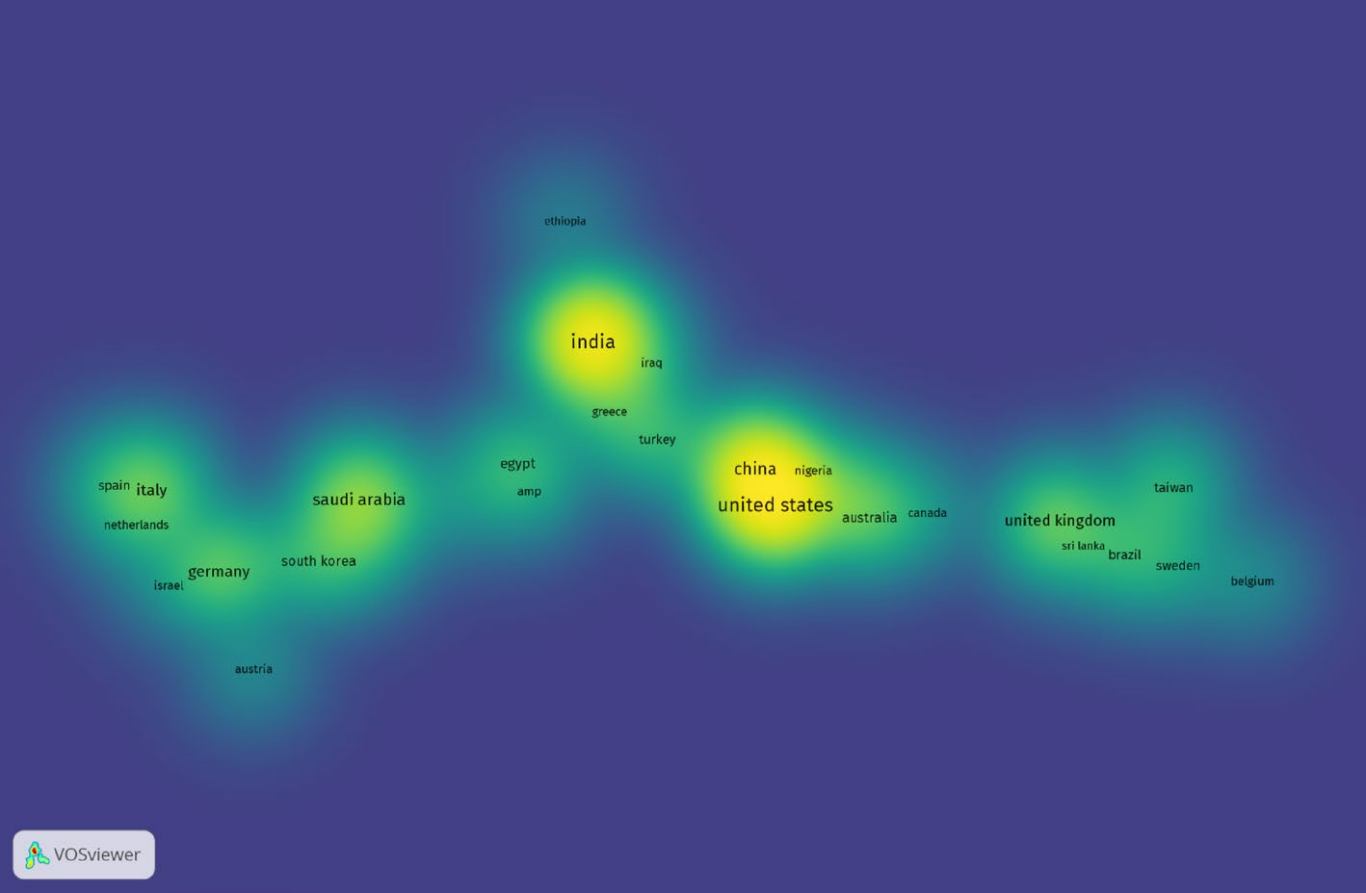
Visual density of citations based on country

We then analyzed the number of publications by country (Fig. 6). The United States of America (USA) has the highest number of citations, followed by India, the United Kingdom (UK), Saudi Arabia, and Brazil. Importantly, the United States, with 59 articles, is the most cited country, appearing in 767 documents (Table S4). Other listed countries have published more than five articles, with fewer than 300 citations. Though the USA is the most cited country in this field of work, by geographical region Europe ranks the highest in number of citations (2366 citations) compared to North America (914 citations) (Table S5 and S6). In short, the USA, India, and the UK lead in publications, underscoring their pivotal role in advancing this research domain. On the other hand, Europe and East Asia (including China) are the two regions with the highest number of publication citations.

### Intellectual structure of author keywords

**Fig. 7:**
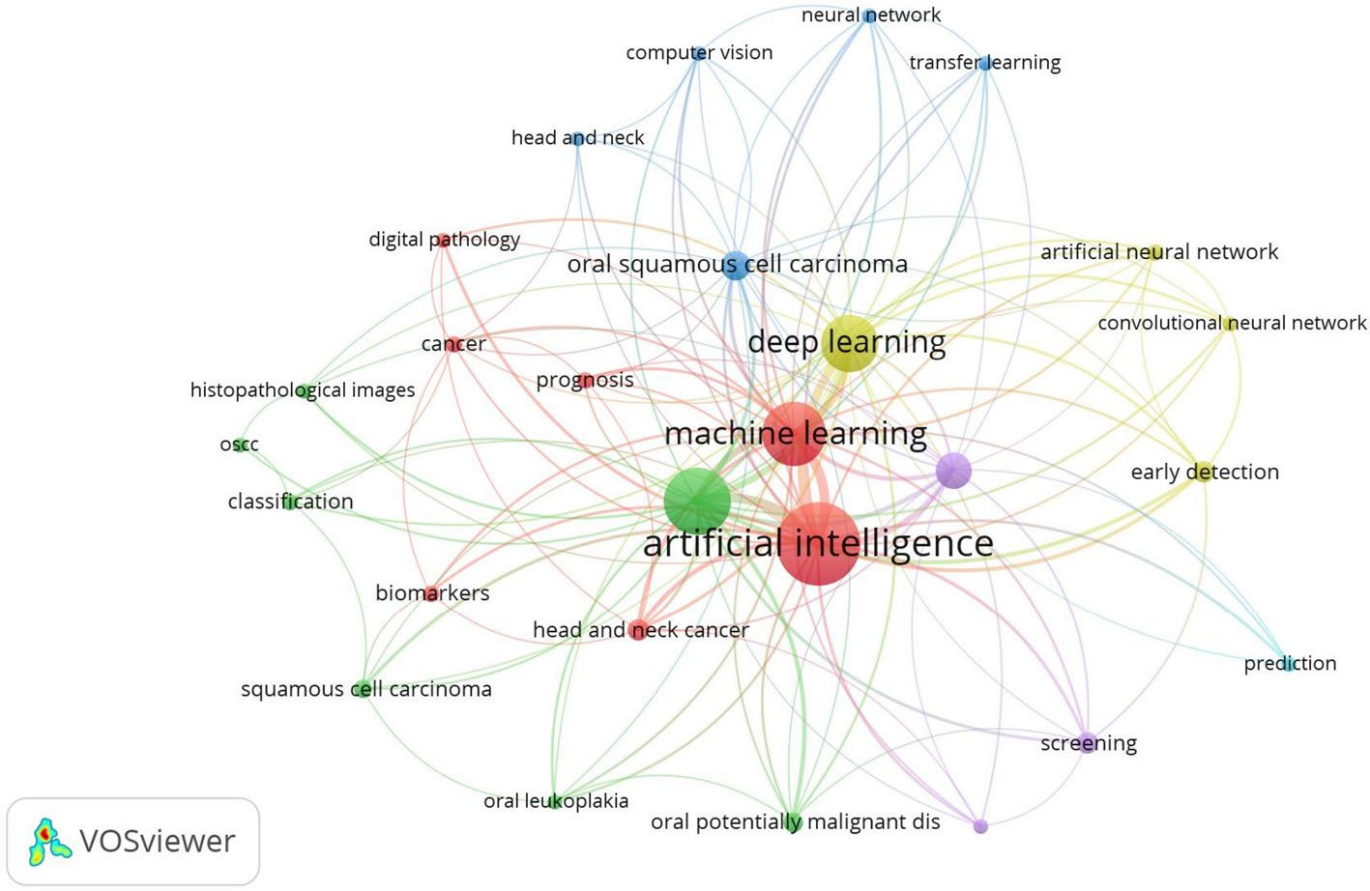
Distribution of research topics in AI related to OC

To develop a co-occurrence network that matches the bibliography, it is necessary to first extract the author’s keywords. Keywords were counted using the full counting technique to avoid duplication and were automatically extracted. In this data analysis, the authors’ keywords were set with a minimum number of occurrences of five, and 27 articles met these criteria. The analysis shows several nodes connecting keywords (Fig. 7). In addition, similar keywords were categorized into clusters (Figure S1). Frequently occurring keywords include ‘AI,’ ‘Deep Learning,’ ‘Machine Learning,’ and ‘Oral Cancer,’ indicating dominant themes in the field.

## Discussion

A bibliometric analysis was performed to obtain a comprehensive overview of research related to AI in OC over the past 30 years. Overall, this field has shown an increasing trend, especially in the last five years, indicating the promise of applying related technological developments to the early diagnosis of OC. This study is expected to provide ideas for researchers to further advance the contribution of AI to OC diagnosis.

Bibliometrics is a method of analyzing literature based on bibliometric parameters such as title, keywords, journal, year of publication, citations, and countries with the most publications [14, 15]. Bibliometrics is widely used to present relationships in research domains by using quantitative methods. Utilizing the same approach as in epidemiology, researchers typically answer questions about a field based on data about publications (e.g., authors, topics, titles), akin to epidemiologists who request patient data to gain an understanding of the health of an area’s population [12, 16]. 

Bibliometrics assists researchers in identifying research ideas, such as in the case of the previously carried out bibliometric analysis in dentistry by Xie et al. [17] This bibliometric analysis showed that several branches of dentistry have begun to use AI to assist clinical procedures. For example, in oral surgery, computer visual technology is used to aid in surgical procedures [17]. AI aims to encourage researchers to further increase their knowledge and develop technologies to support the advancement of dental medical devices [17]. Several studies have performed bibliometric analyses of OC, specifically focusing on the molecular mechanisms of OC [18], risks of OC among people living with HIV/AIDS [19], oral microbiota, and cancer [20], as well as the current trends and themes of OC research [21]. Our study is unique in that it focused on the use of AI in OC to address technological developments in dentistry and the diagnosis of OC. This study included research on AI in OC over 30 years, such as the study by Ilhan et al.., which combined auto-fluorescence and polarization probes with clinical symptoms, habits, and signs and incorporated them into a deep learning-based algorithm to develop an indicator for OC screening [22]. 

Although few studies have focused on the use of AI technology in OC diagnosis, there has been an increase in the number of publications over the last five years in the emerging field of AI applications for the early diagnosis and treatment of OC [14, 23]. The results of our study are expected to encourage further investigations of AI in OC [23, 24]. Analysis of previous research results is necessary for a theoretical and practical understanding of the emerging role of AI in OC. Data research on AI in OC was extracted from SCOPUS and Microsoft Excel, revealing eight major clusters showing collaboration between authors. Using full-counting techniques to prevent data duplication, we extracted the most prevalent keywords used by the researchers. Our analysis highlighted AI, machine learning, deep learning, and oral squamous cell carcinoma as the most frequent topics in AI research on OC. Additionally, the three clusters elucidate various aspects of AI technology, its functionalities, and its predictive capabilities based on article titles. Cluster 1 delves into AI technology for OC diagnosis and features keywords, such as AI, biomarkers, cancer, computer vision, transfer learning, machine learning, digital pathology, deep learning, neural networks, OC, oral squamous cell carcinoma, classification, and prognosis. Cluster 2 discussed the techniques and functions of this technology, focusing on artificial neural networks, early detection, and screening. Finally, cluster 3 illustrates the keyword prediction outcomes.

The development of AI for OC diagnosis is of immense research interest. For example, Ramezani et al.. explained that optical coherence tomography (OCT) can be used for the screening and diagnosis of OC. OCT is an example of AI application for early detection and diagnosis of OC [25]. One limitation of this study is its reliance on the SCOPUS data. Future bibliometric efforts should expand to other databases, such as AI Dimensions and Lens, and prioritize evaluating AI applications in clinical practice for enhanced diagnostic and therapeutic outcomes. Additionally, caution is advised when dealing with unclear journals or research produced by organizations outside academic distribution and publishing channels.

**Fig. 8:**
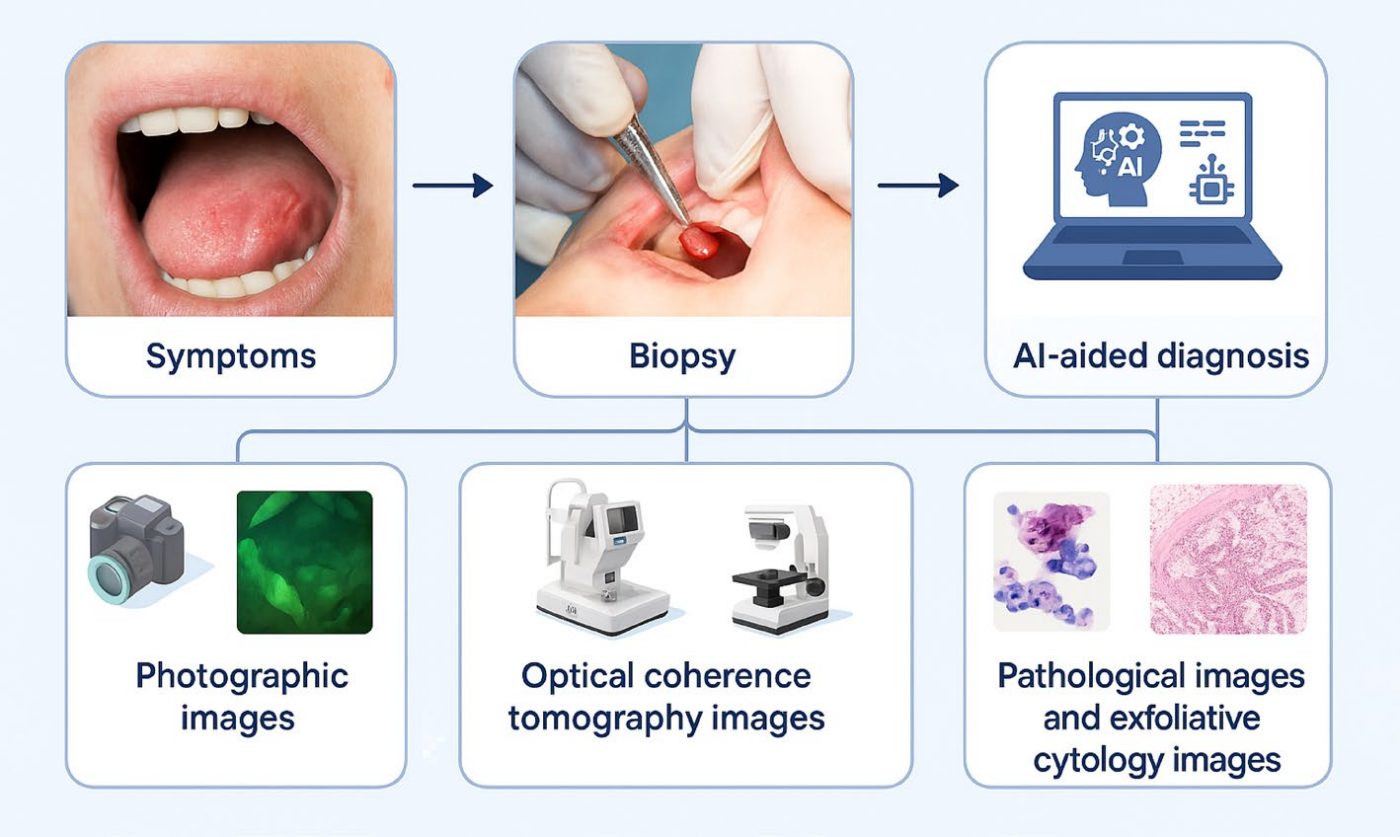
Diagnostic Journey for Oral Cancer with AI Integration. This figure illustrates the diagnostic path for OC, which incorporates AI. The procedure begins with the presentation of clinical symptoms, which is then followed by a biopsy of the suspicious lesions. AI algorithms are used to assess the extracted tissue, as well as numerous diagnostic inputs such as photographic pictures (standard and autofluorescence), optical coherence tomography (OCT) scans, and pathological and exfoliative cytology images. These AI techniques improve diagnostic accuracy by identifying malignancy, categorizing lesion severity, and assisting with prognostic forecasts. AI helps doctors make early, precise, and individualized decisions in the identification and treatment of oral cancer by combining data from several imaging modalities. This diagram was created using Biorender

A critical synthesis of our findings reveals that AI-related research in OC is predominantly concentrated in disciplines such as oral pathology, diagnostic imaging, and oral surgery. These areas have been the most active in integrating AI technologies, particularly in image analysis, lesion classification, and surgical planning (Fig. 8). However, this concentration also underscores a disciplinary gap, with relatively limited contributions from fields like oral medicine and epidemiology, where AI could offer significant value in risk prediction and population-level screening. Furthermore, the geographic distribution of research output reveals a pronounced imbalance, with many high-income countries such as the USA and UK (Table S4) and the European region (Table S5) dominating both publication volume and citation impact. In contrast, there is a striking underrepresentation of research from low- and middle-income countries, despite the high OC burden in some of these regions. This disparity highlights an urgent need to support AI research capacity and infrastructure in underserved settings to ensure equitable development and application of AI tools in global oral health. However, it is important to highlight that India, a lower to middle income country, has the highest number of publications and ranked second in total citations in the field of AI in OC research (Table S4). Moreover, the South Asian region (which includes India) ranks second in the world for total number of publications in the field of AI in OC research (Table S5). This emphasizes the strong uptake of the AI in oral oncology owing to the high burden of OC in that region [1]. 

## Conclusion

In conclusion, our analysis highlights the dynamic growth of AI in OC research. The significant trends in publications and citations reflect the increasing interest and impact of this field. These findings provide valuable insights for policymakers, funding agencies, and researchers, guiding future efforts to integrate AI technologies into oral oncology practices.

## Supplementary Information

Below is the link to the electronic supplementary material.

Supplementary Material 1. Figure S1 Keyword co-occurrence network. Table S1 Top 10 most prolific authors in the field of AI in OC research. Table S2 Top 5 most cited journals in the field of AI in OC research. Table S3 Top ten publications with most citations in the field of AI in OC research. Table S4 Top ten countries based on number of citations in the field of AI in OC research. Table S5 Total number of publications and citations in the field of AI in OC research based on world geographical region. Table S6 Summary of top geographical regions based on total publications and citations in the field of AI in OC research.

## Data Availability

All data generated or analysed during this study are included in this published article [and its supplementary information files].
