# Supplementary material for: A bibliometric analysis reveals a dynamic growth in the use of artificial intelligence in oral cancer research over three decades
Source: Discov Oncol. 2025 Jul 28;16:1432. doi: 10.1007/s12672-025-03293-6 (PMC12304344; doi:10.1007/s12672-025-03293-6)
Supplement: Supplementary file 1 — Supplementary Material 1. Figure S1 Keyword co-occurrence network. Table S1 Top 10 most prolific authors in the field of AI in OC research. Table S2 Top 5 most cited journals in the field of AI in OC research. Table S3 Top ten publications with most citations in the field of AI in OC research. Table S4 Top ten countries based on number of citations in the field of AI in OC research. Table S5 Total number of publications and citations in the field of AI in OC research based on world geographical region. Table S6 Summary of top geographical regions based on total publications and citations in the field of AI in OC research. [file 12672_2025_3293_MOESM1_ESM.docx]

**Supplementary figures and legends**


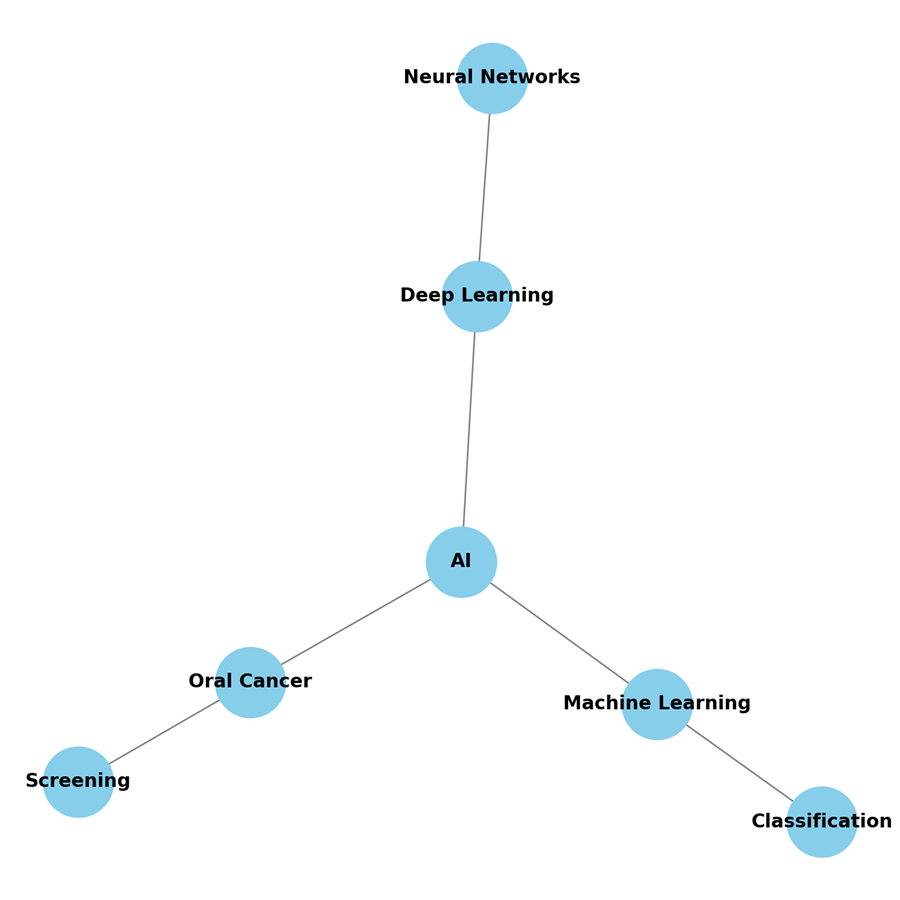


**Figure S1** Keyword co-occurrence network

**Supplementary tables**

**Table S1** Top 10 most prolific authors in the field of AI in OC research.

| Number | Authors | Total publications | TC | CPP | Institution | Country |
| --- | --- | --- | --- | --- | --- | --- |
| 1 | Adeoye J [26,27,28,29,30,31,32] | 9 | 138 | 15.33 | The University of Hong Kong, Hong Kong | Hong Kong |
| 2 | Khurram SA [33,34,35,36,37,38,39] | 8 | 150 | 18.75 | School of Clinical Dentistry, University of Sheffield, United Kingdom | UK |
| 3 | Choi SW [27,28,29,30,31,32] | 6 | 123 | 20.5 | The University of Hong Kong, Hong Kong | Hong Kong |
| 4 | Thomson P [27,28,29,30,31,32] | 6 | 123 | 20.5 | College of Medicine and Dentistry, James Cook University, Cairns, QLD, Australia | Australia |
| 5 | Kerr AR [40,41,42,43,44] | 6 | 38 | 6.3 | New York University College of Dentistry, New York, NY, USA | USA |
| 6 | Kowalski LP [36,37,38,45,46] | 5 | 237 | 47.4 | University of Sao Paulo Medical School, Sao Paulo, Brazil. | Brazil |
| 7 | Makitie AA [45,46,47,48–49] | 5 | 298 | 59.6 | University of Helsinki, Helsinki, Finland | Finland |
| 8 | Alabi RO [46,47,48,49,50] | 5 | 162 | 40.5 | University of Helsinki, Helsinki, Finland | Finland |
| 9 | Elmusrati M [46,48,49,50] | 4 | 153 | 38.25 | School of Technology and Innovations, University of Vaasa, Vaasa, Finland | Finland |
| 10 | Almangush A [45,46,48,49] | 4 | 297 | 53.66 | University of Helsinki, Helsinki, Finland | Finland |

TC = total citations; CPP = citations per publication

**Table S2** Top 5 most cited journals in the field of AI in OC research.

| Number | Journal | The number of publications  (N = 56) | TC | CPP | SJR | Quartile rankings |
| --- | --- | --- | --- | --- | --- | --- |
| 1 | *Cancers* | 19 | 155 | 8.15 | 1.31 | Q1 |
| 2 | *Oral Oncology* | 16 | 367 | 22.9 | 1.27 | Q1 |
| 3 | *Journal of Oral Pathology and Medicine* | 10 | 224 | 22.40 | 0.68 | Q2 |
| 4 | *Diagnostics* | 9 | 113 | 12.55 | 0.67 | Q2 |
| 5 | *Frontiers in Oncology* | 8 | 57 | 7.12 | 1.14 | Q2 |

Notes: TC = total citations; CPP = citations per publication; SJR = SCImago journal Rank; Q = quartile

**Table S3** Top ten publications with most citations in the field of AI in OC research.

| **Number** | **Author** | **Article Title** | **TC** |
| --- | --- | --- | --- |
| 1 | Almangush et al., 2020[45] | Staging and grading of oral squamous cell carcinoma: An update | 144 |
| 2 | Chang et al., 2013 [51] | Oral cancer prognosis based on clinicopathologic and genomic markers using a hybrid of feature selection and machine learning methods | 109 |
| 3 | Ariji et al., 2019 [52] | Contrast-enhanced computed tomography image assessment of cervical lymph node metastasis in patients with oral cancer by using a deep learning system of artificial intelligence | 102 |
| 4 | Majumder et al., 2005 [53] | Support vector machine for optical diagnosis of cancer | 91 |
| 5 | Bur et al, 2019 [54] | Machine learning to predict occult nodal metastasis in early oral squamous cell carcinoma | 87 |
| 6 | Alabi et al., 2020 [46] | Comparison of supervised machine learning classification techniques in prediction of locoregional recurrences in early oral tongue cancer | 79 |
| 7 | Develd et al., 2005 [55] | Autofluorescence and diffuse reflectance spectroscopy for oral oncology | 78 |
| 8 | Exarchos et al., 2012 [56] | Multiparametric decision support system for the prediction of oral cancer reoccurrence | 77 |
| 9 | Ilhan et al., 2020 [22] | Improving Oral Cancer Outcomes with Imaging and Artificial Intelligence | 73 |
| 10 | Sultan et al., 2020 [57] | The use of artificial intelligence, machine learning and deep learning in oncologic histopathology | 64 |

TC = total citations

**Table S4** Top ten countries based on number of citations in the field of AI in OC research.

| **Number** | **Country** | **Number of publications (Articles)** | **TC** |
| --- | --- | --- | --- |
| 1 | USA | 59 | 767 |
| 2 | India | 85 | 576 |
| 3 | UK | 26 | 483 |
| 4 | Saudi Arabia | 28 | 322 |
| 5 | Brazil | 14 | 322 |
| 6 | Finland | 9 | 310 |
| 7 | Sweden | 8 | 309 |
| 8 | Italy | 21 | 260 |
| 9 | Japan | 16 | 241 |
| 10 | China | 36 | 221 |

Notes: TC = total citations

**Table S5** Total number of publications and citations in the field of AI in OC research based on world geographical region.

|  | **South Asia** | **Middle East** | **East Asia** | **Europe** | **North America** | **Latin America** | **Africa** | **Oceania** |
| --- | --- | --- | --- | --- | --- | --- | --- | --- |
|  | India | Saudi Arabia | China | Germany | United States | Brazil | Algeria | Australia |
|  | Pakistan | UAE | South Korea | United Kingdom | Canada | Chile | Egypt | New Zealand |
|  | Sri Lanka | Turkey | Hong Kong | Finland |  |  | Libya |  |
|  | Indonesia | Iran | Taiwan | Italy |  |  | Ethiopia |  |
|  | Malaysia | Iraq | Japan | Sweden |  |  |  |  |
|  | Singapore | Jordan |  | Hungary |  |  |  |  |
|  | Thailand | Israel |  | Slovakia |  |  |  |  |
|  |  | Syria |  | Austria |  |  |  |  |
|  |  |  |  | Switzerland |  |  |  |  |
|  |  |  |  | Belgium |  |  |  |  |
|  |  |  |  | Holland |  |  |  |  |
|  |  |  |  | Romania |  |  |  |  |
|  |  |  |  | Russia |  |  |  |  |
|  |  |  |  | Spain |  |  |  |  |
|  |  |  |  | Czech Republic |  |  |  |  |
|  |  |  |  | France |  |  |  |  |
|  |  |  |  | Greece |  |  |  |  |
|  |  |  |  | Croatia |  |  |  |  |
|  |  |  |  | Denmark |  |  |  |  |
|  |  |  |  | Latvia |  |  |  |  |
| **# of countries** | 7 | 8 | 5 | 20 | 2 | 2 | 4 | 2 |
| **# of publications** | 97 | 59 | 84 | 121 | 60 | 15 | 4 | 6 |
| **# of citations** | 683 | 875 | 1494 | 2873 | 795 | 322 | 7 | 48 |

**Table S6** Summary of top geographical regions based on total publications and citations in the field of AI in OC research.

| **RANK OF TOTAL PUBLICATIONS BASED ON REGION** | **TOTAL PUBLICATIONS** |
| --- | --- |
| 1) EUROPE | 121 |
| 2) SOUTH ASIA | 97 |
| 3) EAST ASIA | 84 |
| 4) NORTH AMERICA | 60 |
| 5) MIDDLE EAST | 59 |
| 6) LATIN AMERICA | 15 |
| 7) OCEANIA | 6 |
| 8) AFRICA | 4 |
| **RANK OF TOTAL CITATIONS BASED ON REGION** | **TOTAL CITATIONS** |
| 1) EUROPE | 2873 |
| 2) EAST ASIA | 1494 |
| 3) WESTERN ASIA | 875 |
| 4) NORTH AMERICA | 795 |
| 5) SOUTH ASIA | 683 |
| 6) LATIN AMERICA | 322 |
| 7) OCEANIA | 48 |
| 8) AFRICA | 7 |
